# Mechanical Properties and Durability of Composite Cement Pastes Containing Phase-Change Materials and Nanosilica

**DOI:** 10.3390/ma17133271

**Published:** 2024-07-02

**Authors:** Javier Ziga-Carbarín, Lauren Y. Gómez-Zamorano, Arquímedes Cruz-López, Soorya Pushpan, Sofía Vázquez-Rodríguez, Magdalena Balonis

**Affiliations:** 1 Programa Doctoral en Ingeniería de Materiales, Facultad de Ingeniería Mecánica y Eléctrica, Universidad Autónoma de Nuevo León, Ave. Universidad s/n, Ciudad Universitaria, San Nicolás de los Garza 66455, Nuevo León, Mexico; gildardo.zigaca@uanl.edu.mx (J.Z.-C.); sooryapuspan@gmail.com (S.P.); sofia.vazquezrd@uanl.edu.mx (S.V.-R.); 2 Departamento de Tecnología del Concreto, Facultad de Ingeniería Civil, Universidad Autónoma de Nuevo León, Ave. Universidad s/n, Ciudad Universitaria, San Nicolás de los Garza 66455, Nuevo León, Mexico; 3 Departamento de Ingeniería Ambiental, Facultad de Ingeniería Civil, Universidad Autónoma de Nuevo León, Ave. Universidad s/n, Ciudad Universitaria, San Nicolás de los Garza 66455, Nuevo León, Mexico; arquimedes.cruzlpz@uanl.edu.mx; 4 Department of Materials Science and Engineering, University of California Los Angeles (UCLA), 410 Westwood Plaza, 2121K Engineering V, Los Angeles, CA 90095, USA

**Keywords:** Portland cement, fly ash, nanosilica, phase-change materials, durability, thermal conductivity, thermal behavior, thermal comfort

## Abstract

Escalating global surface temperatures are highlighting the urgent need for energy-saving solutions. Phase-change materials (PCMs) have emerged as a promising avenue for enhancing thermal comfort in the construction sector. This study assessed the impact of incorporating PCMs ranging from 1% to 10% by mass into composite Portland cement partially replaced by fly ash (FA) and nanosilica particles (NS). Mechanical and electrochemical techniques were utilized to evaluate composite cements. The results indicate that the presence of PCMs delayed cement hydration, acting as a filler without chemically interacting within the composite. The combination of FA and PCMs reduced compressive strength at early ages, while thermal conductivity decreased after 90 days due to the melting point and the latent heat of PCMs. Samples with FA and NS showed a significant reduction in the CO_2_ penetration, attributed to their pozzolanic and microfiller effects, as well as reduced water absorption due to the non-absorptive nature of PCMs. Nitrogen physisorption confirmed structural changes in the cement matrix. Additionally, electrical resistivity and thermal behavior assessments revealed that PCM-containing samples could reduce temperatures by an average of 4 °C. This suggested that PCMs could be a viable alternative for materials with thermal insulation capacity, thereby contributing to energy efficiency in the construction sector.

## 1. Introduction

Population growth and anthropogenic activity have resulted in an approximate 1.5 °C increase in the average global surface temperature over the past 200 years [1,2,3]. The consumption of primary energy on our planet has witnessed a steady rise, with figures showing an escalation from 8588.9 million tons of oil equivalent (Mtoe) in 1995 to 13,147.3 Mtoe in 2015. Projections for 2035 anticipate the production of 17,487 Mtoe [4,5]. In this context, buildings account for about 30% of total energy consumption [6,7,8], with air conditioning, necessary for maintaining thermal comfort, responsible for approximately 20% of global electricity usage [9,10]. In Mexico, the absence of an environmental culture is widespread in conventional building systems. More than 95% of homes lack thermal insulation, and 85% of these buildings are in extremely hot regions, leading to increased energy consumption due to the use of air conditioning [11].

Therefore, it is critical to develop strategies for efficient energy usage, become aware of energy problems, and investigate options to reduce energy consumption and halt this environmental crisis [12]. In the building industry, the utilization of stricter environmental policies to combat climate change has compelled this sector to explore other technological solutions, such as alternative cementitious materials [13,14,15,16], sustainable buildings [17,18], and, nowadays, phase-change materials, which can be added to cementitious materials [19,20]. Unfortunately, while these promising routes are undergoing development, to this date, worldwide energy consumption is still increasing.

Facing new requirements inherent to sustainability, it is crucial that we take advantage of the physicochemical properties of phase-change materials, such as their low melting point, high latent heat, and unique thermal behavior, among others [21,22]. Phase-change materials (PCMs) have been studied since the 1980s, with a focus on improving energy efficiency in buildings [23,24,25,26]. PCMs are sensitive to temperature gradients, producing useful cooling or heating effects upon melting and solidifying at the phase-change temperature. When the temperature increases, a phase transition from solid to liquid occurs, absorbing heat; vice versa, when the temperature drops, the material transitions from liquid to solid, releasing heat [27].

Two types of PCMs have been extensively studied and successfully applied to Portland cement-based materials: inorganic PCMs, mostly salt-hydrate-based, and organic PCMs, primarily paraffin-based. Some advantages of inorganic PCMs include (i) a high heat of fusion and thermal conductivity (almost double that of paraffin), (ii) low volume changes associated with melting, and (iii) cost-effectiveness. However, inorganic PCMs have some disadvantages, including (i) corrosion risk in reinforced concrete, (ii) supercooling, which prevents the release of the latent heat, or (iii) incongruent melting. On the other hand, organic PCMs are known for being safe, reliable, predictable, less expensive, non-corrosive, chemically inert, and stable below 500 °C [25,28,29,30].

Other studies have indicated that micro-encapsulated PCMs, based on paraffins, are very effective if used in the temperature interval of the human comfort zone, from 20 °C to 30 °C [31], and are more cost-effective as compared to the first type. The most recent studies report significant improvements in the development of PCMs used for reducing thermal loads in buildings [32,33,34], but there is a lack of information on durability indicators, particularly those related to aggressive environmental agents [35,36,37,38]. Moreover, it is necessary for these PCMs to have a certain durability, since the presence of these materials could affect reparation processes [39]. Considering this, a decrease in compressive strength upon the addition of high amounts of PCMs has also been reported [37,40,41,42]. In recent studies on Portland cement-based materials, including cement pastes, mortars, and concretes, it has been demonstrated that an increase in the percentage of PCM addition (up to 30% by mass of cement) leads to a subsequent decrease in compressive strength, with reported reductions of up to 50% [43,44]. This reduction in compressive strength can be attributed to a combination of three factors: (i) the addition of a PCM generally increases porosity and consumes water that was meant to participate in hydration reactions, (ii) the bond strength between the PCM and cement paste could be weak, and (iii) the PCM could exhibit low shear strength and stiffness, making it susceptible to rupture under load, possibly creating voids post rupture [45,46]. However, it has also been observed that an increase in PCM percentage results in reduced thermal conductivity values within the range of 0.1 to 0.3 W/mK. These reductions in thermal conductivity can be directly attributed to the inherently low thermal conductivity of PCMs, as well as other factors: (i) PCMs can alter the microstructure of cementitious matrices, (ii) PCMs reduce the density of the material, and (iii) PCMs increase the trapped air content (and air is known to act as a thermal insulator), which benefits cement-based materials in terms of reduced thermal conductivity values in samples containing higher PCM content [47].

Taking all the above into consideration, the main objective of this research was to characterize samples containing 1, 3, 5, and 10% (by mass of cementitious materials) of PCM, 50% of FA, and 1% of nanosilica by means of mechanical strength tests, porosity determinations, and measurements of the depth of carbonation, water adsorption (sorptivity), electrical resistivity, and thermal behavior. Establishing durability indicators for cement matrices containing phase-change materials is essential to ensure the reliability and longevity of these materials when deployed at the industrial scale. Such indicators allow us to have confidence in the expected lifespan and maintenance requirements of these structures. This research aims to offer viable solutions for constructing durable homes and buildings in regions experiencing extreme heat, specifically in Mexico, by utilizing ordinary Portland cement, fly ash, nanosilica, and PCMs. The main objective is to enhance thermal comfort, thereby reducing energy consumption and promoting sustainable development.

## 2. Materials and Methods

**Figure 1:**
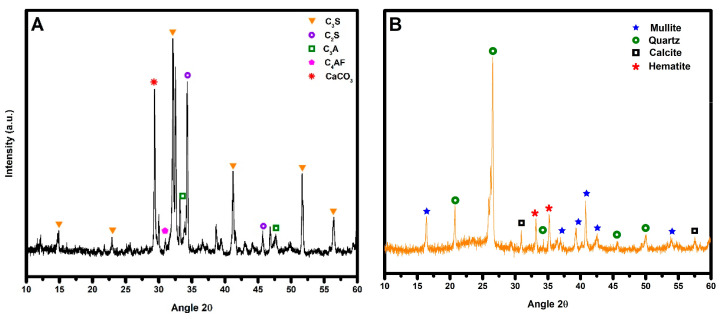
X-ray patterns of raw materials. (**A**) OPC and (**B**) FA.

The materials utilized in this investigation were as follows: (a) Ordinary Portland cement (OPC-40) from CEMEX, Monterrey, N.L., México, with a density of 3.06 g/cm^3^, and (b) fly ash (FA) class F, provided by a thermo-electric plant in Nava, Coahuila, Mexico, characterized by a particle size below 75 μm and a density of 2.03 g/cm^3^. Table 1 presents chemical composition determined by X-ray fluorescence (XRF) analysis. Figure 1 shows X-ray diffraction (XRD) patterns collected for both raw materials to ascertain phases present in OPC and FA. Nanoparticles employed in this study were commercially available as AEROSIL MOX 170, comprising 98.3%wt of SiO_2_, with the remaining mineral component being Al_2_O_3_. The PCM utilized was Micronal 24D acquired from Microtek Laboratories Inc; Moraine, OH, USA. It is an acrylic PCM, appearing white to slightly off-white in color, and with individual particle sizes of 3 μm, protected by an organic polymer shell based on polymethyl methacrylate. It exhibits a melting point of 24 °C ± 2 °C and the latent heat of 97 J·g^−1^.

In this investigation, the impact of PCM addition on the OPC, with and without the presence of FA, was examined, following in the footsteps of other authors [48]. In both sample types, the addition of 1% silica nanoparticles was evaluated in terms of enhancing nucleation sites and promoting compressive strength development, as designated by mix proportion shown in Table 2. The same water-to-cement (w/c) ratio was maintained, since it has been reported that the PCM behaves as a non-absorbent material, similar to graded quartz sand [35]. Consequently, no significant reduction in workability was observed across all mixtures. Before mixing, powders were homogenized in a mixer at a speed of 180 ± 5 rpm. The nanosilica (NS) was pre-dispersed in the mixing water for 5 min at the same speed. A similar procedure was reported elsewhere [16].

To achieve this, cubic samples measuring 2.5 cm were prepared and initially cured for 24 h at room temperature, followed by further curing under a Ca(OH)_2_ saturated solution for up to 90 days [35]. A semi-adiabatic process was assumed to monitor the temperatures of all the samples during the initial 24 h of curing. Temperature readings were taken one minute after the completion of the mixing process. To facilitate this, a 2 cm deep hole was created in one face of the cube to insert K-type thermocouples, which were connected to a 24-bit data acquisition card (NI 9211). Electrical signals were recorded every 2 s and processed using National Instruments LabView 2018 software.

After hydration, samples underwent characterization through compressive strength tests, involving the extraction of four specimens from the containers, with the average value then reported. Subsequently, pieces of the solid fractions from the sample centers were crushed and immersed in isopropyl alcohol for 24 h, followed by drying in an oven at 50 °C for an additional day to stop hydration reactions prior to characterization [49,50]. Post-drying, samples were ground to obtain a powder with a particle size below 75 μm. Qualitative X-ray diffraction (XRD) analyses were conducted on the powdered samples utilizing a Bruker-D8 Advance diffractometer with Cu-Kα radiation (λ = 1.54). Samples were scanned on a rotating stage within a 10° to 60° 2θ range, with a step size of 0.021 and a dwell time of 1 s. The acquisition time for X-ray diffraction patterns averaged around 35 min. X-ray structure data for known compounds were sourced from the literature or standard databases (ICD, JCPDS). For scanning electron microscopy (SEM) analyses, specimens were cold-mounted in epoxy resin, ground, polished, and gold-coated, prior obtaining backscattered electron images. The equipment employed was a JEOL model JSM 6510LV by JOEL USA, Inc. Peabody, MA, USA.

The measurements of thermal conductivity were conducted using a Linseis Transient Hot Bridge THB 1 apparatus and an isolated Kapton sensor model THB6K58. Samples measuring 30 × 50 × 5 mm from P and FA series were fabricated and subsequently analyzed after 7, 28, and 90 days of curing. However, before each analysis, samples were kept at 60 °C in the oven until constant weight was achieved, ensuring a variation in mass of ≤0.2%. The conditions for thermal conductivity measurements were set at 0.1 A for 39 s. Physisorption analyses were carried out using an Autosorb-1 apparatus by Quantachrome, employing 0.5 g of various samples cured for 7, 28, and 90 days. Each sample was placed in a 9 mm glass cell, securely sealed, and then subjected to degassing at 20 °C for 24 h to eliminate impurities and absorbed water. Subsequently, each sample underwent vacuum treatment to initiate nitrogen physisorption via automatic pulses until reaching P/P° = 1. The obtained absorption/desorption isotherms provided insights into the type, shape, and diameter of pores. Carbonation was assessed in samples cured for up to 90 days. Samples were prepared following a modified procedure according to BS EN 13295 [51], wherein two faces of each sample were coated with epoxy paint while leaving four faces exposed. These samples were then placed inside a chamber (model FFCO500RTABB by FISHER) for 7 days in an atmosphere with 4% CO_2_, a relative humidity of 60% ± 10%, and a temperature of 25 °C. After the specific time had elapsed, samples were fractured and sprayed with a phenolphthalein indicator solution, prepared from 1 g of colorant, 70 mL of ethanol, and 30 mL of deionized water.

Electrical impedance analysis was conducted on samples cured for 7, 28, and 90 days using a Solartron 1287 potentiostat and galvanostat coupled with an Analytical 1260 Solartron frequency response analyzer and ZPLOT software version 3.5i. The experimental setup involved a 3-electrode arrangement [52], with electrical pulses applied in a scan of 90 points at frequency intervals ranging from 3 MHz to 100 Hz, utilizing a 50 mV excitation wave source. Obtained spectra were analyzed using ZVIEW software version 3.5i and fitted to equivalent circuit models to calculate electrical resistivity. Sorptivity tests were performed following a modified ASTM C1585 procedure [53] to determine the initial and final adsorption rates. After 90 days of curing, samples were dried at 50 °C for 3 days and then placed in a dryer containing a potassium bromide solution. Each sample was sealed in a hermetic container for 15 days at the temperature of 23 °C ± 2 °C. Subsequently, five faces of the sample were sealed with a vinyl tape, leaving one exposed face in contact with water, and weight changes were recorded over an 8-day period, reaching an error of 0.1 g.

**Figure 2:**
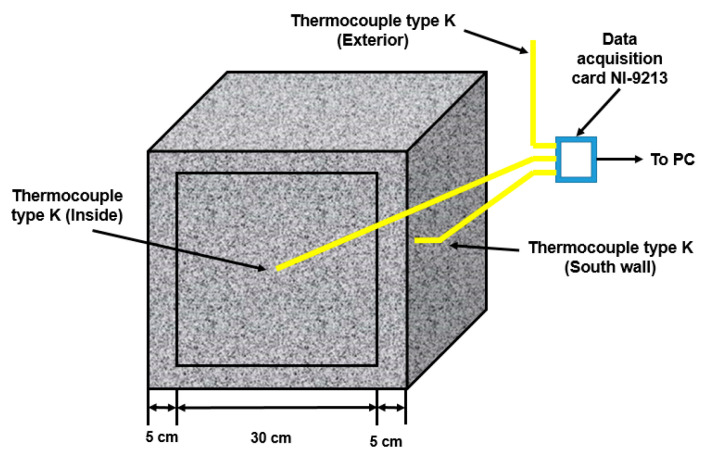
Experimental arrangement used for the measurement of thermal behavior.

To assess thermal behavior, mixtures with the lowest thermal conductivity values (FA3 and FA10) were selected (as presented in the Results section), since this property remained constant after 90 days of curing in water. Additionally, reference (R) and FA0 samples were examined. For each sample, a box measuring 30 cm from wall to wall, with a wall thickness of 5 cm, was constructed (Figure 2). The box was exposed to external conditions for 7 days, with its sides facing south, aided by a digital compass. K-type thermocouples were positioned on the south face and at the center of the box. The four experiments were conducted simultaneously using National Instrument’s 24-bit 16-channel data acquisition card NI-9213. The card was programmed to take measurements every 10 s, and, with the assistance of LabView 2018 software, acquired data were averaged every 30 min.

## 3. Results and Discussion

### 3.1. Early-Stage Temperature Measurements

**Figure 3:**
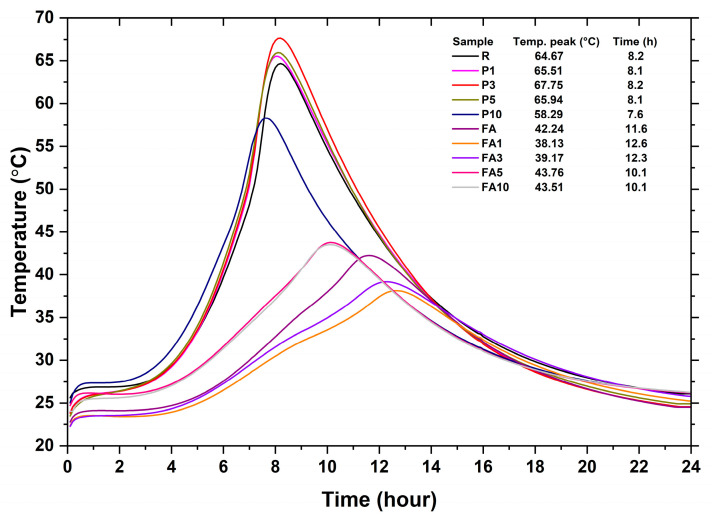
Hydration temperatures recorded during the first 24 h.

Figure 3 displays results of the semi-adiabatic hydration peak temperature measurements for all the samples studied at early ages, similar to those that other authors have reported [54]. Obtained results indicate that the reference R sample and its corresponding P series (P1, P3, and P5) exhibited similar peak temperatures, near 60 °C. In sample P10, a reduction in the maximum temperature and time was observed (by 6.38 °C and 0.6 h, respectively), probably due to the intrinsic capacity for heat adsorption and thermal conductivity in the PCM which modified the hydration rate and process. For fly ash (FA)-containing samples, FA1 and FA3 required more than one hour to reach the maximum hydration temperature, which was 10% slower than that recorded for FA0. In contrast, samples FA5 and FA10 achieved their maximum temperature one hour faster, due to the thermal conductivity and adsorption properties of the PCM [55,56]. From the collected data, it was observed that specimens with the highest PCM content reduced the heat of hydration without retarding the hydration reaction rate. However, other authors reported a reduction of 3 °C in maximum hydration temperature when 5% of PCM was added in a ternary mixture of ordinary Portland cement, fly ash, and slag [32,47]. Additionally, Naser P. Sharifi et al. reported an 8 °C reduction in maximum hydration temperature in mortars, attributing the results to the percentage of PCMs embedded in the pastes leading to more homogeneous heat absorption and reduction in the melting point of the PCMs [32]. However, in this work, while the temperatures measured were slightly different, the overall trends observed were like those reported elsewhere [57,58,59]. Thermal behavior trends can be associated with the intrinsic characteristics of PCMs, which can promote the nucleation and growth of products, thereby accelerating the hydration of C_3_S and C_2_S and the overall dissolution and precipitation of cement minerals [60].

### 3.2. Compressive Strength

**Figure 4:**
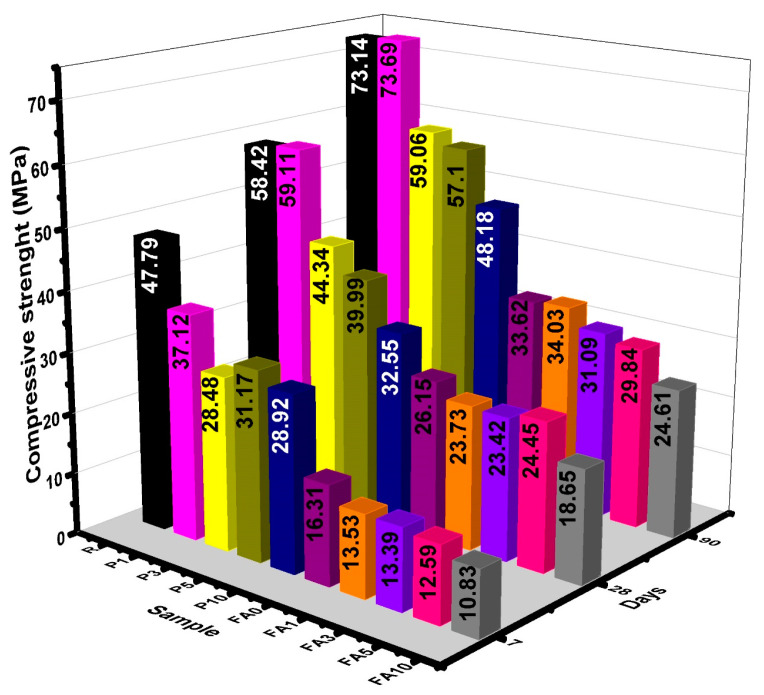
Compressive strength data for all the samples cured at 7, 28, and 90 days.

Figure 4 illustrates results of the compressive strength measurements. At 7 days, sample P1 exhibited an 11.17% reduction in compressive strength as compared to the reference sample R, while, at 28 and 90 days, there was no significant difference found. Sample P3 showed a decrease in the compressive strength by 31.83%, 24.10%, and 19.25% at 7, 28, and 90 days, respectively. Similarly, sample P5 experienced reductions of 25.4%, 31.54%, and 21.92% for the same ages, and, for P10, a similar trend was observed, with compressive strength decreasing by 30.78%, 44%, and 34.12% at 7, 28, and 90 days, respectively. These results indicate that samples with a PCM content greater than 3% exhibited a decrease in the compressive strength between 30 and 35%. This reduction in compressive strength in specimens with paraffin-based PCMs present in their mixtures is attributed to multiple factors, as previously reported [47], with the following ones highlighted: (i) PCMs function as voids/gaps in the specimens [58], (ii) the nature of the bonds at the interface between the cement grains and PCM particles could be weak due to the hydrophobic nature of PCMs [61], and (iii) adding a higher percentage of PCMs to specimens reduces the cement content. Similar studies have attributed the aforementioned factors to a reduction in strength in cement-based composites containing graphite encapsulating PCMs and nanosilica [46,62].

In the case of the FA series, the addition of PCMs showed similar trends to the ones described above. Sample FA1 exhibited decreases in compressive strength by 17.9%, 10.43%, and 7.52% at 7, 28, and 90 days, respectively. Similarly, the assessed reduction for sample FA5 was recorded at 22.8%, 6.5%, and 11.24%. With an increase in the PCM addition (sample FA10), the diminution of compressive strength was even higher, with 33.59%, 28.68%, and 26.79% drops recorded at 7, 28, and 90 days. These reductions are not significant when compared to FA0; however, when compared to R (the reference sample), there is a significant decline in compressive strength, due to the high quantity of FA that could not completely react with the CH present in cement, hence hindering the pozzolanic reaction [62,63]. The observed reductions in compressive strength were lesser as compared to other reports, where a reduction of 40% has been attributed to the presence of voids in paste caused by the low affinity between PCMs and binders [64]. Moreover, it has also been reported that with higher levels of PCM addition, the low strength and stiffness of the PCM will also contribute to a reduction in the overall strength [65]. Even though reductions were observed, samples presented structurally acceptable compressive strength from day 28 onwards. In cases when nanosilica (NS) is included, several reports have indicated that in percentages lower than 2%, NS could affect the interfacial transition zone (ITZ) between the PCM and the cement paste and possibly trigger the formation of calcium silicate hydrates, which could fill the gap within the ITZ [46,66]. The use of nanoparticles in cement pastes, employing numerical analytical methods to predict mechanical stresses, has been reported elsewhere [67]. By considering variables such as the volume fractions of the filler and matrix, these methods can be applied to understand the impact of PCM addition on mechanical properties such as compressive strength.

### 3.3. X-ray Diffraction

**Figure 5:**
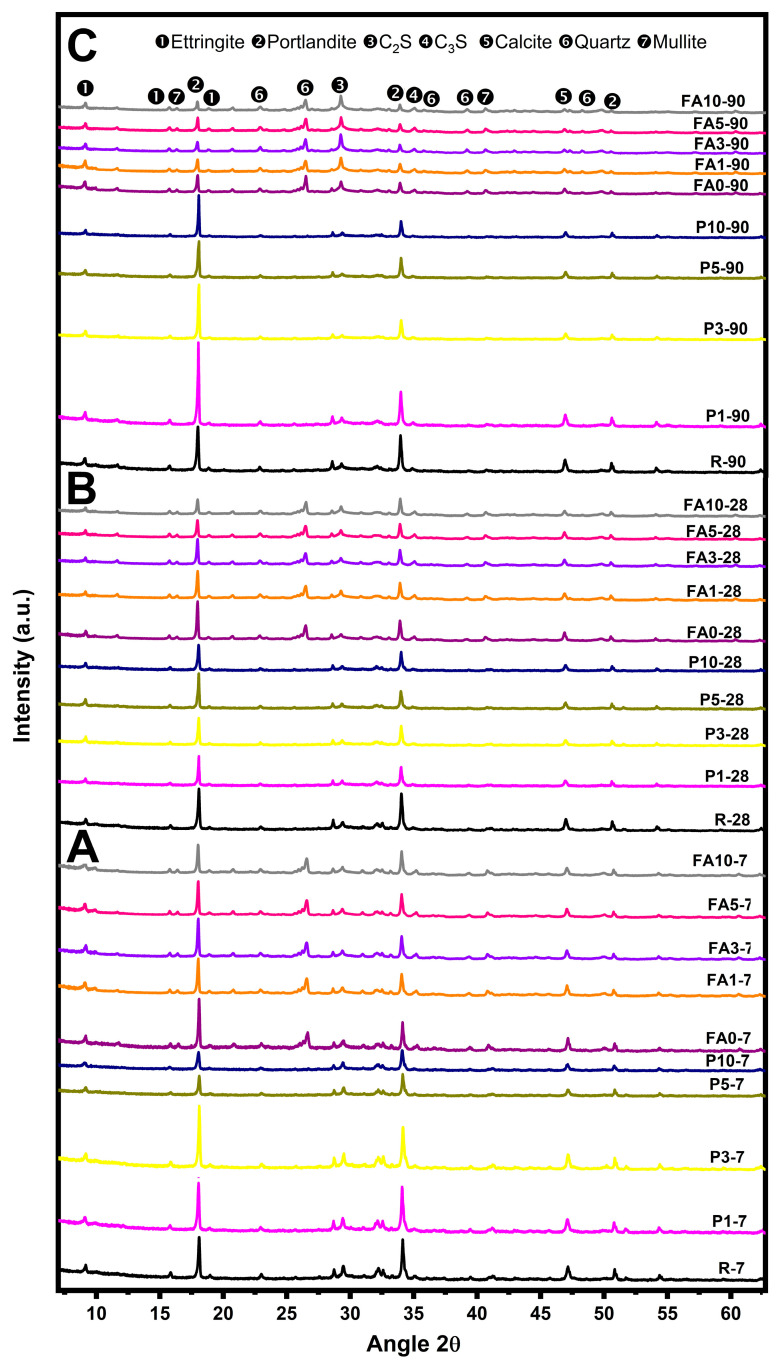
X-ray diffraction patterns of pastes: P and FA series (**A**–**C**) at 7, 28, and 90 days, respectively.

The X-ray diffraction patterns obtained at 7, 28, and 90 days are shown in Figure 5. Samples cured at 7 days (Figure 5A) exhibited reflections characteristic of Portland cement: portlandite at 2θ angles of 18.089°, 28.662°, 34.089°, 50.795°, and 54.337° (according to ICDD card 00-004-0733); C_2_S at 29.356°, 32.054°, and 50.079° (ICDD card 00-0333-0306) [68]; C_3_S at 32.705° and 33.305° (ICDD card 00-037-1476), and calcite at 39.425° and 43.22° (ICDD card 01-080-2791). However, the presence of a high amount of PCM reduced the intensity of these peaks. Ettringite at 2θ = 9.05°, 15.773°, and 22.899° (ICDD card 00-017-0445) was also observed in the XRD patterns [68,69,70]. Samples without FA cured at 28 days showed that pastes made with PCMs exhibited a greater intensity in their portlandite reflections.

In fact, at 90 days, all samples of the P series showed higher intensity of portlandite peaks (2θ = 18.089°) when compared to the reference sample R (Figure 5C). Samples containing FA, in addition to the previously mentioned phases, revealed the presence of quartz identified at 2θ = 20.827°, 26.607°, 36.489°, 39.429°, and 50.079° (ICDD card 01-009-8935) [69,70] and mullite at 2θ = 16.428° and 40.875° (ICDD card 01-074-4144). The addition of the PCM and FA caused a decrease in the intensity of portlandite (2θ = 18.089° and 34.089°) and calcite (2θ = 39.425° and 43.22°) reflections, a phenomenon which could be related to the dilution effect and an overall delay in the hydration reactions. At 28 and 90 days, the CH consumption was evident in all samples containing FA, indicating that the pozzolanic reaction had taken place at those curing times. The results were consistent with those reported elsewhere, where the reported addition of fly ash in cement pastes showed a reduction in calcium hydroxide (CH) content [71,72,73].

### 3.4. Scanning Electron Microscopy

**Figure 6:**
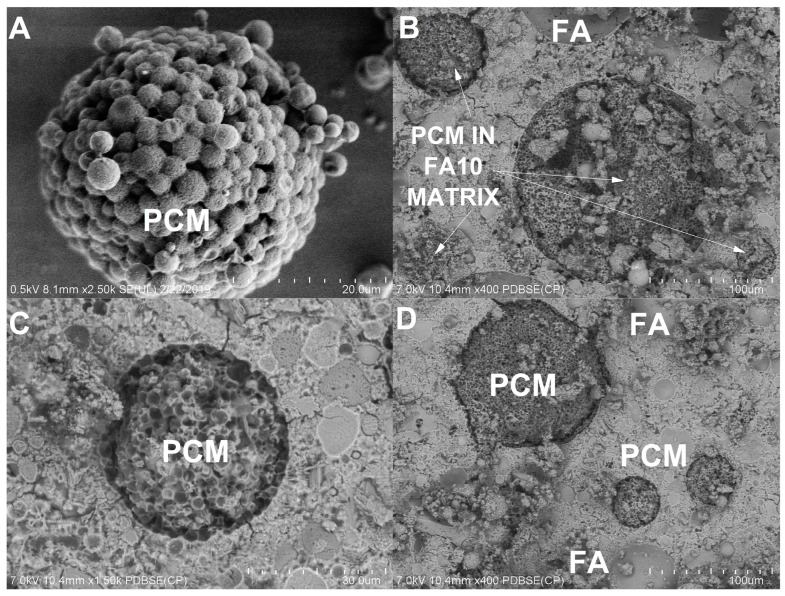
Scanning electron microscope images of (**A**) the PCM and (**B**–**D**) sample FA10.

Images obtained via scanning electron microscopy (SEM) for sample FA10 cured at 90 days are presented in Figure 6. Analyzing Figure 6A, it is notable that PCM particles consist of individual spheres with a diameter of approximately 3 μm, which could form spherical agglomerates with an average size ranging from 50 to 170 μm, as shown in the SEM images. In Figure 6B–D, images of the PCM particles that had occluded in the FA10 paste after 90 days are depicted. The PCM spheres retained their physical structure, confirming that PCMs are resistant to physical manipulation and do not react with the cementitious phase. The presence of unreacted FA particles can also be observed in the upper part of the micrograph. Additionally, in this image, the filler effect of the PCM in the cementitious matrix is apparent. The number of fractured particles increased depending on the PCM content, since PCMs are the not reactive bonding agents in cement and have low stiffness [65].

### 3.5. Thermal Conductivity

**Figure 7:**
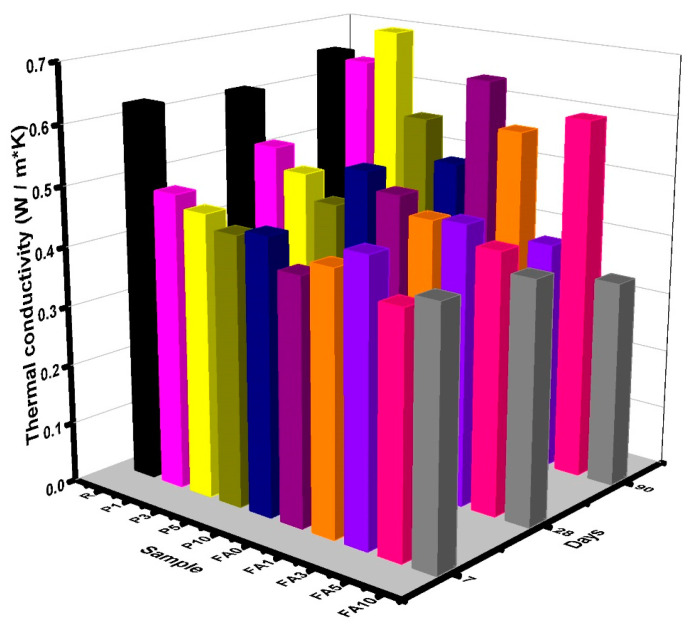
Thermal conductivity results obtained for P and FA series at 7, 28, and 90 days.

The thermal conductivity results obtained for dry samples are presented in Figure 7. At 7 and 28 days, a reduction in values was observed for samples P1 and P3, by 22% and 16%, respectively, while, at 90 days, their values were very close to that measured for the reference sample (R). A similar trend was observed for sample FA5. Reductions at early ages can be directly attributed to the values of the melting point and latent heat characteristic for the PCM materials, and, at later ages, they can be directly linked to the microstructural modifications caused by the addition of PCMs, meaning that the heat penetrated at the same speed as in the R sample [74]. When the addition of the PCM increased from 5% to 10% by mass (P5 and P10 samples), these values decreased when compared to R by an average of 22%. Several authors have indicated that the presence of an interfacial gap between the PCM particles and the cement paste reduces thermal conductivity. Olivieri et al. investigated the use of a PCM in a cementitious matrix, adding it at intervals from 20 to 32%, and obtained thermal conductivities ranging from 0.93 to 1.27 W/m∙K. The PCM used in their study had a melting point of 26 °C and latent heat of 100 kJ/kg [47,74]. The reduction in thermal conductivity can also be attributed to the fact that the thermal conductivity of paraffins, such as those used in this research, vary from 0.2 to 0.4 W/m∙K [75]. Meanwhile, for cement pastes, thermal conductivities of 0.53–1.38 W/m∙K have been reported [76]. Nevertheless, in this work, while the values recorded were slightly lower, the behavior in terms of thermal properties was similar. The presence of FA also reduced these values, with additions greater than 3% showing a decrease of nearly 47% at 90 days as compared to FA0. When PCMs were incorporated into a geopolymer, the specific heat capacity increased significantly, which demonstrated that PCMs could reduce heat transport through the geopolymer matrix. This could open up the possibility for PCMs to be used as a construction material that could enhance thermal behavior [65].

### 3.6. Nitrogen Physisorption

**Figure 8:**
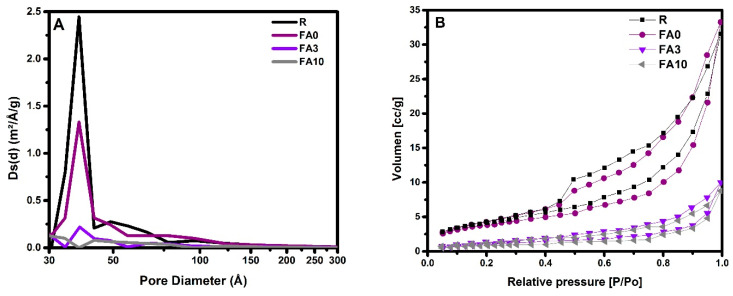
Nitrogen physisorption results obtained for samples FA3, FA10, FA0, and R at 90 days. (**A**) Pore diameter and (**B**) absorption–desorption isotherms.

The nitrogen physisorption results for samples FA3, FA10, FA0, and R cured at 90 days indicated a specific surface area of 4.19, 3.08, 14, and 15 m^2^/g, respectively, showing a decrease in the specific area due to the addition of PCMs (Figure 8). Figure 8A presents the pore size distribution versus the volume, where samples FA3 and FA10 have low porosity in the interval of 30 to 100 Å (3 and 10 nm), while reference samples showed bimodal distribution at the same interval. As a result, PCM addition modified the porous structure of the cement matrix, and it is also likely that the nanoparticles affected the tortuosity of the samples, acting as a filler material. The impact on the tortuosity of the mixtures is observed in the nitrogen physisorption results, specifically in the change of pore type, and in the reduction of CO_2_ penetration, which suggests a change in the pore interconnection of the matrices. Regarding hysteresis graphs (Figure 8B), reference samples correspond to H3, where the absorption and desorption lines indicate a different porosity. FA3 and FA10 showed type H4 isotherms, which correspond to micro-mesoporous structures [77]. These behaviors could be attributed to the fact that the incorporation of PCMs can shift pore size distribution values towards smaller numbers as compared to the samples without PCMs, since PCMs can fill a certain type of pore in the cementitious matrix [78].

### 3.7. Depth of Carbonation

**Figure 9:**
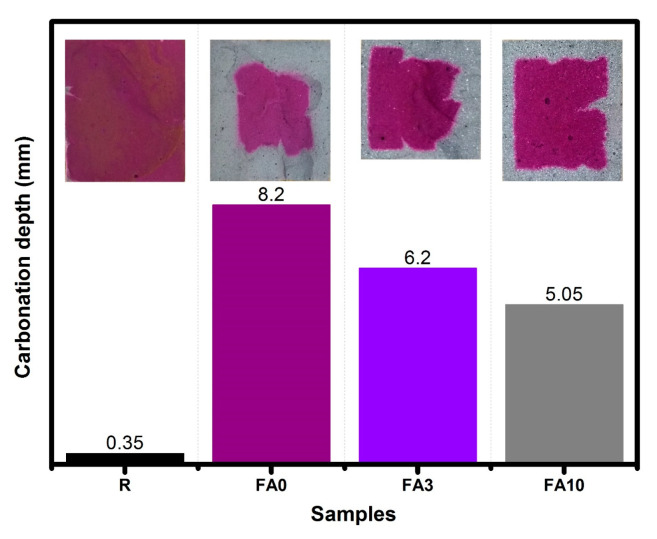
CO_2_ penetration depth for recorded samples FA3, FA10, FA0, and R at 90 days.

A reduction in CO_2_ penetration was observed in the range of 24% and 38% for FA3 and FA10 when compared to FA0 (Figure 9). Samples FA3, FA10, FA0, and R, when cured for 90 days, revealed CO_2_ penetration depths for the exposed faces as follows: 6.2, 5.05, 8.2, and 0.35 mm, respectively. While these results do not fully align with the pore size distribution, they are potentially associated with the fact that closed pores could have been affected in the cementitious matrix upon the addition of PCMs [79]. Additionally, due to a similar filler effect, as reported for nanosilica particles, PCMs could act as a barrier and help to reduce the penetration of corrosive agents by filling pores, as shown in the SEM results (see Figure 6C). Furthermore, the use of silica particles helps to reduce the depth of carbonation due to the pozzolanic reaction and the filler effect, which modify the overall porosity of the cement matrix [80,81].

### 3.8. Electrical Resistivity

**Figure 10:**
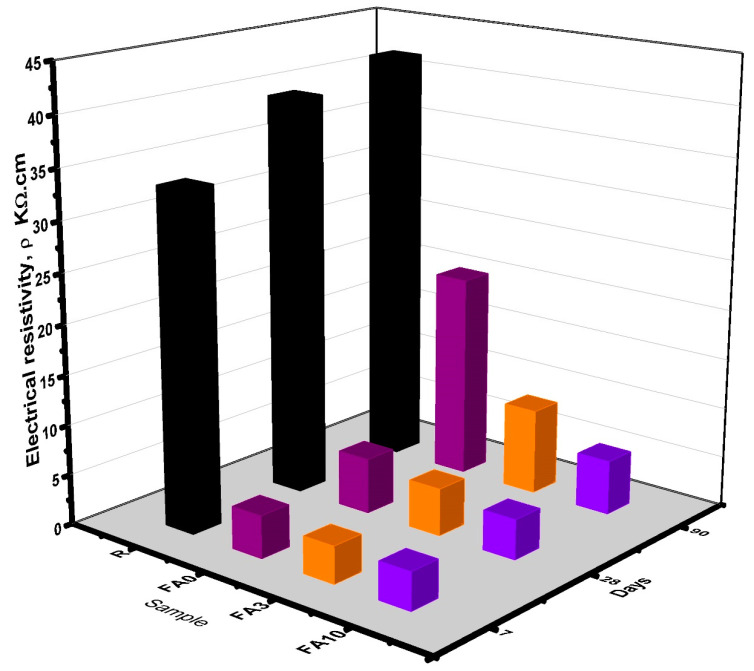
Electrical resistivity results obtained for samples FA3, FA10, FA0, and R at 7, 28, and 90 days.

Electrical resistivity in cement pastes depends on the pore solution composition and porosity; if these factors change within a cementitious matrix, then the electrical resistivity will change substantially, as reported elsewhere [67]. The electrical resistivity results obtained for samples FA3, FA10, FA0, and R at 7, 28, and 90 days of curing are shown in Figure 10. In general, samples exhibited an increase in electrical resistivity as time elapsed. However, samples FA3 and FA10 showed a reduction in electrical resistivity of 79.57% and 87%, respectively, as compared to the reference sample (R). These values are close to the recommended electrical resistivity threshold, which assures a better protection of structures containing reinforcing steel [52]. From the results, it is evident that PCMs provide both filler and barrier effect.

### 3.9. Water Absorption (Sorptivity)

**Figure 11:**
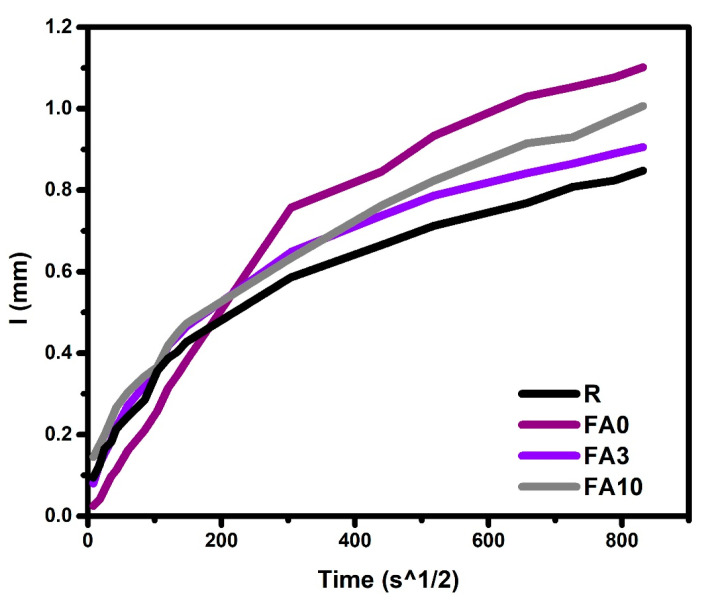
Sorptivity analysis for samples FA3, FA10, FA0, and R at 90 days.

Sorptivity results obtained for samples FA3, FA10, FA0, and R at 90 days of curing are presented in Figure 11. In the first 6 h, it was noted that samples FA3 and FA10 yielded results slightly higher than those obtained for FA0 and R, and, during this time, the average absorption rate of 0.0024 mm/s^1/2^ (Table 3) was recorded. This could be attributed to the non-absorbent behavior of the PCM [35]. After this time had elapsed, a change in the final absorption rate, mainly in sample FA0, was detected (Table 4). This could be linked to the porosity of the cementitious matrix, which could have prevented more interconnected pores from arising, as corroborated by the nitrogen physisorption results. On the other hand, the incorporation of silica nanoparticles can help to reduce absorbed water, as reported by other authors [80,81].

### 3.10. Thermal Behavior

**Figure 12:**
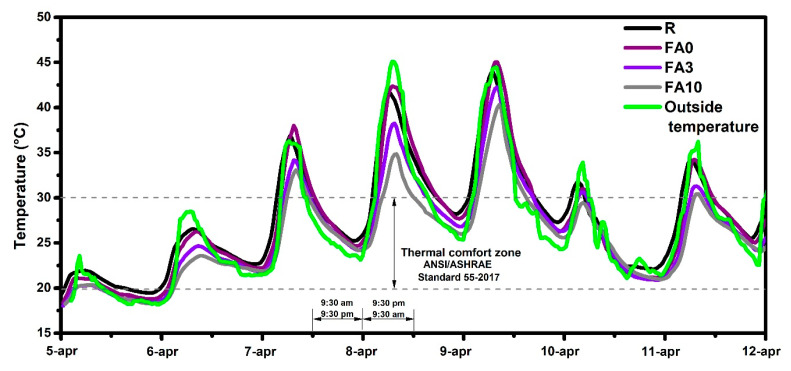
Thermal behavior of boxes exposed for 1 week to the outside environment.

This section discusses thermal behavior of samples FA3, FA10, FA0, and R when exposed to the outside conditions for 1 week, with the temperature ranging from 25 to 45 °C (Figure 12). For 5 days, the maximum temperature values were kept above the comfort zone specified by the ANSI/ASHRAE Standard 55-2017 [82]. In general, it was noted that samples with the PCM percentages above 3% reduced the temperature on average by 4 °C when compared to the ambient temperature, similar to what has been reported elsewhere [83,84]. These results support the claim that the physicochemical properties of PCM-embedded cementitious samples are altered and that the PCM helps to minimize heat transport through the cement matrix.

**Figure 13:**
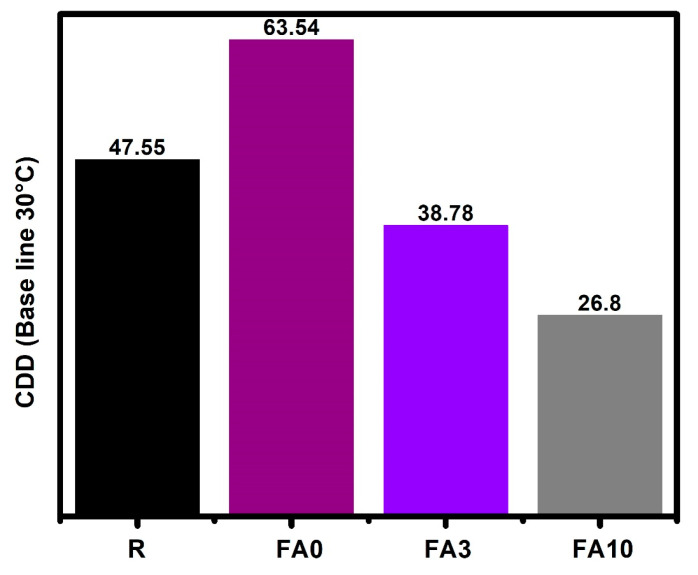
Cooling degree days with respect to the baseline set at 30 °C.

Analyzing cooling degree day (CDD) correlations according to ANSI/ASHRAE Standard 55-2017 [82] and using 30 °C as a baseline (Figure 13), samples FA3 and FA10 showed a reduction of nearly 37% in comparison to FA0. These results indicate that the temperature inside the boxes was reduced in the presence of PCMs.

Moreover, this means that when the temperature exceeds 30 °C, the conventional materials used to build our homes and buildings (represented in this work by R and FA0) would require 47.55 and 63.44 CDDs to maintain the thermal comfort zone. With an increase in the PCM percentage addition, a reduction in CDDs was observed, i.e., for box FA3, 38.78 CDDs were needed to reach the thermal comfort zone, and for box FA10, 26.8 CDDs were required to reach the thermal comfort zone in buildings. This means a reduction of 58% in terms of CDDs as compared to R. These findings are similar to those reported by other authors [85].

## 4. Conclusions

Analyzing the impact of PCM addition on composite Portland cement blends containing fly ash, the following conclusions were drawn:

1. The temperatures recorded by K-type thermocouples during the semi-adiabatic process in the fresh pastes, with data collected every 2 s, revealed that adding 10% PCMs by the mass of cement reduced the hydration time and temperature at early ages. This reduction is attributed to the PCM’s heat absorption capacity and intrinsic thermal conductivity properties.
2. SEM images confirmed that PCM particles have individual sizes of 3 μm and tend to agglomerate in spherical shapes ranging from 50 to 170 μm in diameter. These spheres fill spaces in the cementitious matrix, physically altering microstructure and porosities. These modifications compromise certain properties, such as compressive strength. It was found that adding 10% PCM led to a reduction of nearly 33% in strength. Additionally, electrical resistivity values drastically decreased when the PCM content exceeded 3%, with a reduction of 87% as compared to the reference sample (R). These decreases confirmed that the PCM modified the microstructure acting as microfiller, hence altering the electrical resistivity, and these observations were supported by the SEM results.
3. For samples cured up to 90 days, deep carbonation measurements were conducted by exposing samples to a controlled atmosphere for 7 days, followed by fracturing and spraying them with a phenolphthalein indicator solution. Samples exhibiting lower thermal conductivity (FA3 and FA10) showed lower depths of carbonation in the presence of the PCM, hence reducing risk of penetration by aggressive agents.
4. Another improved property was water absorption. Samples dried for 3 days and exposed to water demonstrated that PCMs behaved like non-adsorbent materials. Specifically, FA3 exhibited better performance in terms of the final absorption rate, while for FA samples, the initial absorption rate was improved.
5. Regarding thermal behavior, temperature measurements obtained by K-type thermocouples during 7-day exposure to the external conditions indicated that PCM addition could potentially enhance thermal comfort within buildings. Consequently, the use of PCMs in combination with high-fly-ash-containing Portland cement blends present a viable option for thermal insulation and, by extension, the mitigation of global warming.

## Figures and Tables

**Table 1:** Chemical analyses of OPC-40 and fly ash, with values obtained through XRF measurements.

	Oxide (%)											
	SiO_2_	Al_2_O_3_	Fe_2_O_3_	CaO	MgO	SO_3_	Na_2_O	K_2_O	TiO_2_	P_2_O_5_	MnO	LoI
**OPC**	19.82	4.97	1.99	61.47	1.48	4.53	0.5	0.81	0.24	0.1	0.04	0.04
**FA**	59.86	24.30	3.98	2.72	0.78	0.42	0.54	0.54	0.86	0.04	0.01	0.04

LoI: Loss on ignition.

**Table 2:** Mix proportion of cement pastes with and without the PCM, using the w/c ratio of 0.45 (675 g).

Components	R	P1	P3	P5	P10	FA0	FA1	FA3	FA5	FA10
Cement (gr)	1500	1500	1500	1500	1500	750	750	750	750	750
Fly ash (gr)	--	--	--	--	--	750	750	750	750	750
PCM (gr)	--	15	45	75	150	--	15	45	75	150
Nanosilica (gr)	--	15	15	15	15	--	15	15	15	15

**Table 3:** Initial absorption rates.

Sample	Lineal Regression up to 6 h	R^2^	Si (mm/s^1/2^)
**R-90**	y = 0.0023x + 0.0971	0.98	0.00234
**FA0-90**	y = 0.0026x + 0.0026	0.99	0.00255
**FA3-90**	y = 0.0027x + 0.0877	0.98	0.00267
**FA10-90**	y = 0.0023x + 0.1482	0.98	0.00225

**Table 4:** Final absorption rates.

Sample	Lineal Regression from Day 1 to Day 8	R^2^	Sf (mm/s^1/2^)
**R-90**	y = 0.0005x + 0.4488	0.99	0.00049
**FA0-90**	y = 0.0007x + 0.5685	0.98	0.00066
**FA3-90**	y = 0.0027x + 0.0877	0.98	0.00047
**FA10-90**	y = 0.0007x + 0.4537	0.98	0.00067

## Data Availability

The original contributions presented in the study are included in the article, further inquiries can be directed to the corresponding authors.
